# A Specific and Sensitive Aptamer-Based Digital PCR Chip for *Salmonella typhimurium* Detection

**DOI:** 10.3390/bios12070458

**Published:** 2022-06-26

**Authors:** Yuanjie Suo, Weihong Yin, Qiangyuan Zhu, Wenshuai Wu, Wenjian Cao, Ying Mu

**Affiliations:** 1 Research Centre for Analytical Instrumentation, Institute of Cyber-Systems and Control, State Key Laboratory of Industrial Control Technology, Zhejiang University, Hangzhou 310027, China; 11832043@zju.edu.cn (Y.S.); wenjian.cao@zju.edu.cn (W.C.); 2 College of Life Sciences, Zhejiang University, Hangzhou 310058, China; 22007060@zju.edu.cn (W.Y.); wenshuai.wu@zju.edu.cn (W.W.)

**Keywords:** sensitive detection, aptamer, selective enrichment, *S. typhimurium*, digital PCR chip

## Abstract

Food poisoning and infectious diseases caused by *Salmonella typhimurium* (*S. typhimurium*) are serious public health concerns for human health and food safety. The diversity and complexity of food matrices pose great challenges for rapid and ultra-sensitive detection of *S. typhimurium* in food samples. A method capable of identification, detection, and quantification of *S. typhimurium* is essential for addressing these issues. In this study, aptamer-coated magnetic beads (Apt-MBs) are employed as capture bio-probes to specifically and selectively concentrate *S. typhimurium* in food samples. A self-priming chip-based digital PCR was then presented as another biosensor for on-site detection and quantification of *S. typhimurium* cells. The chip we developed was robust and did not require any external power for sample loading. The combination of Apt-MBs with an on-chip digital detection realized the integration into lab-on-a-chip-based biosensors for on-site monitoring of foodborne pathogens. It was possible to capture and detect *S. typhimurium* cells as low as 90 CFU/reaction with a capture efficiency of 94.5%. Additionally, the whole process only took about 2 h. This unique platform could also be used to monitor other target bacteria with high specificity and sensitivity by utilizing different aptamers. Furthermore, the platform has potential applications in point-of-care testing in the future.

## 1. Introduction

*S. typhimurium*, one of the most representative foodborne pathogens, has been classified as an important indicator for food safety monitoring and analysis. It can lead to human salmonellosis disease [1,2] and its clinical features include fever, vomiting, serious diarrhea, bacteremia, and abdominal cramps within 8–72 h [3,4]. *S. typhimurium* can infect a variety of poultry meat products and human beings through transmission vectors, such as contaminated water and food [5,6], thus, posing a threat to human health. It has been estimated that *Salmonella* spp. are responsible for 93 million foodborne diseases and 155,000 deaths globally every year [7]. More than 75% of the *Salmonella* outbreaks were related to contaminated food, including seed vegetables, eggs, chicken, pork, beef, or vegetable crops [8]. Therefore, the monitoring of *S. typhimurium* in food is of great significance for human health. A conventional bacterial culture method based on bacterial growth in media is the gold standard method with high accuracy. However, it usually takes a few days (5–7 d) to obtain results and requires an enrichment step followed by selective plating and biochemical and/or serological identification [8,9]. Up to now, several polymerase chain reaction (PCR) approaches are developed as an alternative for the rapid detection of *S. typhimurium* in complex food samples [10]. Additionally, it features short response times, high throughput, and high sensitivity. However, a pre-enrichment step is still inevitable for most samples, especially for complex food samples. Food inhibitors, such as salts, preservatives, and a variety of microbiota, may interfere with the detection steps. To increase the sensitivity, specificity, and rapidity of the detection method, a pre-analytical sample preparation step is important in order to separate, concentrate, and purify the target bacteria from the food samples.

Recently, aptamers have attracted considerable attention for the detection of pathogens due to their advantages, including specificity, reproducibility, and ease of modification. Aptamers are single-stranded DNA or RNA that can bind to specific targets by folding into complex three-dimensional structures [11]. Wang et al. [12] employed specific aptamers as capture molecules to enrich *S. typhimurium* cells. In addition, in some aspects, aptamers have major advantages over antibodies. They are not sensitive to virus or bacterial pollution, which reduces the production cost. They also have a good practicability for sensors and actuators [11]. Besides, food substrates are usually complex and diverse, which makes it particularly important to isolate and purify target bacteria from complex food samples. The existing microflora can often interfere with the identification results and limit the sensitivity of the detection [13]. Magnetic separation provides a good way for the separation and purification of bacteria in complex food matrices [14,15]. Hence, the combination of magnetic beads and aptamers can better play the roles of capture, separation, and purification. However, the single aptamer-based detection method has some limitations in the accuracy of the detection. Other techniques are required to assist in the detection of the target bacteria.

Microfluidics has received an extensive attention and is frequently used for biological detections. It has outstanding advantages, such as small size, less reagent consumption, high reaction efficiency, low cost, and point-of-care diagnosis [16,17,18]. More importantly, microfluidic chips can be combined with a variety of technologies, such as digital PCR (dPCR), loop-mediated isothermal amplification (LAMP) and mass spectrometry, and optical-based techniques [19], which provides convenience for the integrated detection systems in the future. Although mass spectrometry-based detection methods can rapidly detect multiple bacteria, they need to rely on proteomic approaches or spectra matching [20,21,22]. Most of the optical-based bacterial detection methods need to train a variety of standard strains without labels in advance and rely on the use of antibodies [23,24,25]. There are high requirements for optical devices. Furthermore, these methods have many difficulties in the process of integrated development with chips. In comparison, digital PCR has better specificity and sensitivity in detecting bacteria by amplifying specific gene sequences [26]. Moreover, the digital PCR technique can be combined with a microfluidic chip to achieve absolute quantification, and it is suitable for point-of-care testing (POCT). Microfluidic chips supply a reliable platform for dPCR. Yin et al. [27] developed a self-priming dPCR chip to perform a digital multiplex detection of major foodborne pathogens and realized “sample-in–answer-out”. Yu et al. [28] developed a self-partitioning SlipChip to perform dPCR and digital melting curve analysis for pathogen detection. Although these digital chips exhibited an accurate quantitative performance that is highly valuable for POCT, they are not suitable for complex food substrates.

Herein, we designed a novel platform to meet the demand for rapid analysis and a simple strategy suitable for the POCT detection of pathogens. It involves Apt-MBs for the specific capture and enrichment of target *S. typhimurium* cells and a self-priming chip that identifies and detects the target *S. typhimurium* cells. Apt-MBs were employed for specific aptamer-targeted bacteria recognition. The concentrated samples were then automatically driven into the chip by negative pressure in order to carry out the subsequent dPCR reaction. The entire workflow was evaluated, including the sensitivity, specificity, and capability of testing real samples.

## 2. Materials and Methods

### 2.1. Materials and Apparatus

Polydimethylsiloxane (PDMS) was purchased from Momentive Performance Materials (Waterford, NY, USA). A negative photoresist (SU-8 3050) and a developer were obtained from MicroChem (USA). Trimethylchlorosilane was purchased from Sigma-Aldrich (St. Louis, MO, USA). Streptavidin-coated superparamagnetic nanobeads (100 nm) were purchased from Nanjing NanoEast Biotech Co., Ltd. (Nanjing, China). Luria-Bertani (LB) medium and tryptone soya agar (TSA) were from Qingdao Haibo Biotechnology Co., Ltd. (Qingdao, China). A PCR mix and RNase-free water were purchased from ThermoFisher Scientific (USA). The primers and probes were synthesized by Sangon Biotech (Shanghai, China). Tween-20 was purchased from Sigma Aldrich (St. Louis, MO, USA). An Olympus fluorescence microscope (IXplore) was purchased from OLYMPUS (Japan), which was employed for fluorescence imaging.

### 2.2. Cell Culture and Preparation for Food Samples

*S. typhimurium* (ATCC 14028) was obtained from the American Type Culture Collection (ATCC). The strain was cultured in a Luria-Bertani (LB) broth at 37 °C for 18 h. Following the incubation, 8 mL of the enriched culture was pooled into sterile centrifuge tubes and centrifuged at 3000 rpm, at 25 °C for 10 min. The cell pellets were suspended in sterile normal saline (0.85% NaCl) to obtain concentrations ranging from 10^1^ to 10^6^ CFU/mL.

Minced pork meats were purchased from a local supermarket. The samples were processed according to China’s food safety national standards with a slight modification. A total of 25 g of the meats was added into 125 mL of sterile phosphate-buffered saline (PBS), followed by homogenization for 10 min using a stomacher (BagMixer CC, Interscience, Paris, France). The mixed minced pork meat samples were filtered separately using filter stomacher bags (BagPage^®^R, Interscience, Saint Nom la Brétèche, France). The debris-free food matrix solutions were carefully collected. The mixed meat matrices were preserved at 4 °C for further use. Then, different concentrations of *S. typhimurium* were added into the food matrix solutions to prepare spiked pork meat samples with bacterial concentrations from 1.0 × 10^1^ to 1.0 × 10^6^ CFU/mL.

### 2.3. Synthesis of Apt-MBs

The sequence of the *S. typhimurium* aptamer (ssDNA) [29] is 5′-biotin-TATGGCGGCGTCACCCGACGGGGACTTGACCTTGACATTATGACAG-3′. The aqueous solution of streptavidin-modified magnetic beads (5 mg/mL, 5 μL) was incubated with a biotin–aptamer (10 μM, 100 μL) for 1 h at 37 °C. The excessive biotin–aptamer in the supernatant was removed by magnetic separation and the obtained Apt-MBs were resuspended in 500 μL PBS (pH = 7.4) after washing 3 times with PBS to serve as stock solutions. The obtained Apt-MBs stock solutions were stored at 4 °C. Then, the working concentration was obtained by diluting the Apt-MBs stock solution ten-fold.

### 2.4. Optimization of Apt-MBs and the Estimation of Capturing Efficiency

A total of 80, 100, 120, and 140 μL of Apt-MBs was added to the reaction mixture, respectively, and 20 μL of targeted bacterial solution was added and the final volume of the reaction mixture was adjusted to 200 μL with PBS. The mixture was incubated at 37 °C for 30 min and concentrated using a magnetic stand. The supernatant was collected and 100 μL of 1:10 dilution was plated on an agar plate in duplicates and incubated overnight at 37 °C. The concentrated Apt-MBs–*Salmonella* complex was washed 3 times with 1 mL of PBS. A total of 100 μL of each washing solution was plated directly onto NA plates. The efficiency of the Apt-MBs to capture *S. typhimurium* cells was estimated by a bacterial culture colony count (CFU) method. The experiment was repeated three times.

### 2.5. Chip Design and Fabrication

The microfluidic chips in this work were fabricated by soft lithography techniques and PDMS molding techniques. The patterns for the microchannels and microchambers were designed with AutoCAD 2019 (AUTODESK, San Rafael, CA, USA) and printed as a mask. Figure S1 shows schematic illustrations of the fabrication process. First, a silicon wafer was cleaned and baked at 200 °C for 15 min to dehydrate the surface. Then, a 50 μm layer of a SU-8 3050 negative photoresist was applied by spin-coating for 30 s at 1750 rpm onto the clean silicon wafer. The coated wafer was then baked at 95 °C for 15 min and exposed to collimated UV light through a film mask with a channel pattern. The UV-exposed silicon wafer was baked on a temperature-controlled board at 65 °C for 1 min, 95 °C for 15 min, and cooled down to room temperature naturally. Afterwards, the microchamber layer was made by following the above steps. Finally, the wafer was developed in a SU-8 developer at room temperature and baked on a hot plate at 200 °C for 30 min.

The mold was treated with trimethylchlorosilane for 20 min before making the chip in order to promote the peeling of the PDMS. Then, a total of 30 g of PDMS (A:B/10:1) was poured onto the mold and baked at 90 °C for 2 h. Afterwards, the cured PDMS was peeled off from the mold and cut into suitable pieces. Prior to the bonding, inlet and outlets ports were created in the channel layer using a puncher. Then, the PDMS chip was bonded to the glass substrate by a plasma treatment.

### 2.6. Operation and Evaluation of Digital PCR Chip

The operation of the chip mainly uses negative pressure to complete the injection. First, the chip surface was pasted with HD scotch tape to form a relatively sealed environment. Then, the chip was placed in a vacuum tank and degassed to 0.1 kPa for 40 min to exhaust the air inside the PDMS. After that, the adhesive tape on the inlets was punctured with a syringe needle, followed by inserting a pipette tip with the reaction mix. The reaction mix and mineral oil were introduced into the microfluidic chip by self-priming. Then, the inlet and outlet were sealed with the mixture of PDMS (A:B/10:1) and Pt catalyst (10 μL). Finally, the sealed chip was placed on a flatbed thermocycler block (MGL96G, Long Gene) for subsequent PCR reactions.

In order to evaluate the reliability and sensitivity of the dPCR in the chip, a standard curve of *S. typhimurium* detection was generated by the dPCR method. The primers and probes targeted the *invA* genes of *S. typhimurium* [30]. (Forward primers: GTTGAGGATGTTATTCGCAAAGG, Reverse primers: GGAGGCTTCCGGGTCAAG, Probe: 5′-FAM-CCGTCAGACCTCTGGCAGTACCTTCCTC-BHQ-3′.) For the quantification of *S. typhimurium*, each 20 μL of the PCR solution contained the following: 10 μL of PCR mix, 0.5 μL of 100 nM forward primers, 0.5 μL of 100 nM reverse primers, 0.3 μL of taqman probe, 2 μL of template DNA, 1 μL of 0.1% Tween-20, and 5.7 μL of RNase-free H_2_O. The thermocycling protocol for the *Salmonella invA* quantification included a 5 min hot start at 95 °C and 40 cycles of two-step PCR (95 °C for 15 s and 60 °C for 30 s). The experiment was repeated three times.

### 2.7. Evaluation of the Performance of the Proposed Platform

To test the performance and sensitivity of the proposed platform, serial dilutions of the *S. typhimurium* cells were spiked in 200 μL of 10 mM PBS to give final concentrations ranging from 10^2^ to 10^6^ CFU/mL in triplicates. Then, the pathogen samples were concentrated using the optimized Apt-MBs. After washing with the PBS, the concentrated samples were lysed using a cell lysis buffer. The cell lysis process contained 2 steps. First, the cells were lysed in 5 μL of lysis buffer (400 mM KOH, 100 mM DTT, 2 mM EDTA) at 65 °C for 10 min and 85 °C for 10 min. Then, 5 μL of stop solution (600 mM Tris-HCl (pH = 7.5), 400 mM HCl) was added to neutralize the lysis buffer. After briefly spinning down, the lysed cells can be stored at −80 °C for future use. The subsequent dPCR was conducted as mentioned above. The experiment was repeated three times.

### 2.8. Detection of S.Typhimurium in Food Samples Using the Proposed Platform

The spiked pork meat samples with unknown concentrations of *S. typhimurium* cells were prepared as mentioned above. Then, the amount of bacteria in the samples was measured using the plate counting method to evaluate the applicability of the proposed platform for the real samples. The experiment was repeated three times.

### 2.9. Image Acquisition and Analysis

After thermocycling, fluorescence images of the chips were acquired by a fluorescence microscope (IXplore, OLYMPUS, Japan) through a 10× lens (emission: 518 nm; excitation: 494 nm). The calculation of the number of fluorescent points in the fluorescence images was conducted by Image J (NIH Image, MD, USA). The correlation analysis and t-test were conducted by OriginPro 8. 0.

## 3. Results and Discussion

### 3.1. Overview of the Selective Magnetic Enrichment-Based dPCR Platform for S. typhimurium Testing in Complex Food Samples

The detection of foodborne pathogens in food samples is very important, and molecular-based detection techniques have the advantage of high accuracy. However, multiple steps are required before testing, including food matrix separation, pathogen enrichment, and complex DNA extraction steps, resulting in a long time to get the results from the samples [31]. In addition, the performance of the assays can also be affected by complex sample matrices, limiting their practical field applicability [8]. Therefore, there is a continuous requirement for sensitive and robust pre-analytical sample processing tools that will facilitate the separation and enrichment of the target samples for subsequent detections. These methods must reduce sample volumes and remove matrix-associated inhibitors with high recovery of the target. Sample preparation has become a critical step in improving complex matrix management and obtaining a better process throughput. In this direction, magnetic separation was reported as an ideal sample enrichment approach, which is compatible with downstream molecular analysis [32,33,34,35]. Methods based on magnetic beads may simultaneously reduce the matrices‘ effects and enrich the pathogens, thereby increasing analytical sensitivity and specificity. Vinayaka et al. used antibody-modified magnetic beads to capture *S. typhimurium* in foods and performed a direct qPCR reaction [8]. However, direct qPCR has strict requirements for reaction conditions and instruments, limiting their application in on-site diagnosis. Yang et al. constructed a multivalent brush-like magnetic nano-platform for *Listeria monocytogenes* detection with a low detection limit [34]. However, this method targeted Gram-positive bacteria and had certain limitations in capture specificity.

**Figure 1:**
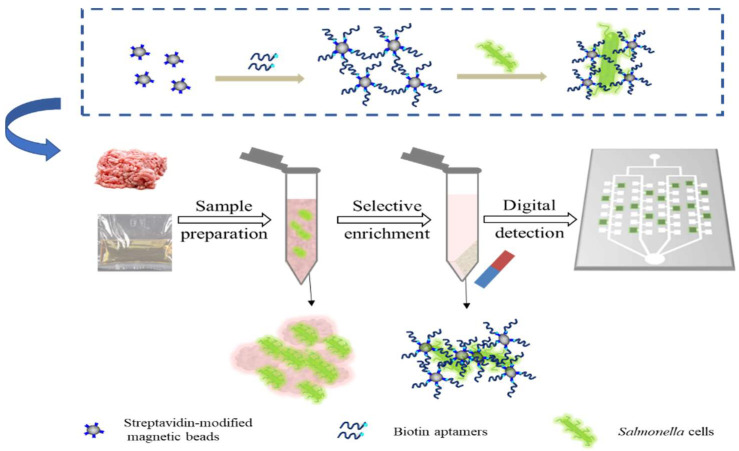
A schematic illustration of selective enrichment and fluorescence detection of *S. typhimurium*.

Our selective magnetic enrichment-based dPCR platform was realized through a dPCR assay implemented within a selective aptamer. The overview scheme is shown in Figure 1. The specificity of this platform comes from two aspects. First, the aptamer-based enrichment removes impurities in the food during the capture. The aptamer binds specifically to the outer membrane protein of *S. typhimurium*, allowing for a precise discrimination of the viable bacteria from the dead ones [36]. This step specifically concentrates the target bacteria, and the volume of samples is reduced to a few microliters. Second, the subsequent dPCR reaction ensures the sensitivity and accuracy of the detection. The application of the chips enables the detection to be integrated into microfluidic devices, which provides a certain basis for the construction of the integrated capture and detection device.

### 3.2. Sensitivity and Capture Efficiency of Apt-MBs

To obtain a high capture efficiency of the Apt-MBs, the amounts of Apt-MBs were optimized with 10^5^ CFU/mL of *S. typhimurium*. Next, 80, 100, 120, and 140 μL of Apt-MBs were added to the mixture, followed by a capture efficiency measurement. The capture efficiency was calculated by the following formula:

(1) $$\mathrm{Capture}\;\mathrm{Efficiency}=\frac{\mathrm{Bacteria}\;\mathrm{concentration}\;\mathrm{in}\;\mathrm{total}-\mathrm{Bacteria}\;\mathrm{concentration}\;\mathrm{left}}{\mathrm{Bacteria}\;\mathrm{concentration}\;\mathrm{in}\;\mathrm{total}}\times 100\%$$

**Figure 2:**
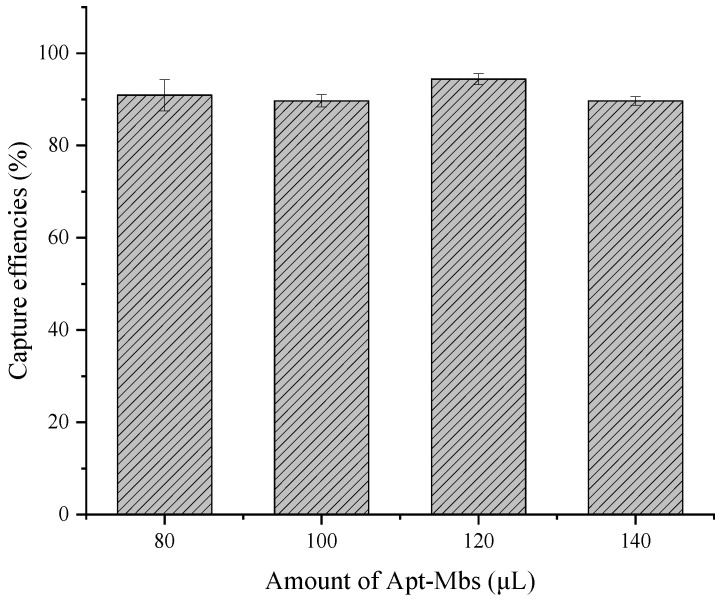
Optimization results for the capture efficiencies of the Apt-MBs. The error bars represent the standard deviation of three replications, *n* = 3.

The results are shown in Figure 2. We performed an optimization experiment of the capture efficiency under 80 μL in the early stage and the results showed that the capture efficiencies were all below 85%. Additionally, it was found that as the concentration increased, the capture efficiencies increased (S2). In order to improve the capture efficiency, we finally settled on 80 μL as the starting point for the optimization experiment. It can be seen that all of these four groups can achieve a capture efficiency of about 90%, which is already high. In addition, the capture efficiency of the four groups is very close. However, the optimization process of the capture efficiency is also affected by the operation, which leads to certain errors. The differences in capture efficiency among the four groups may be a result of the operation deviation. The purpose of the optimization experiment is to find a more stable group as the optimal condition. In addition, during the experiment, it was necessary to avoid wasting the Apt-MBs as much as possible. The 120 μL of Apt-MBs resulted in the highest capture efficiency of 94.5%. Thus, considering the stability and the capture efficiency, the 120 μL of Apt-MBs was selected for the following experiments. The high capture efficiency reduces the loss of bacterial samples, ensuring the low detection limit of the subsequent digital PCR reactions.

### 3.3. Chip Fabrication and Performance of Loading and Sealing

**Figure 3:**
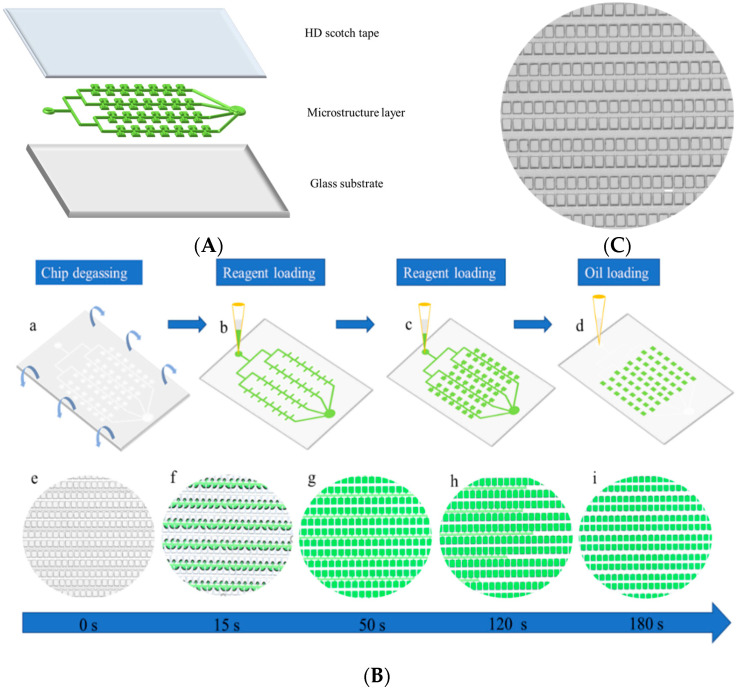
(**A**) A schematic diagram of the layered structure of the microfluidic chip. (**B**) Sample loading of the microfluidic chip. (**a**–**d**) are the schematic diagrams of the sample loading. (**e**–**i**) show the loading process of a green dye in the microfluidic chip. The use of the green dye made the loading process visual. (**C**) A picture of the digital PCR reaction solution being injected into the chip. The microchambers were filled with the reaction solution and the oil phase completely separated the microchambers and the channels. The scale bar is 100 μm.

As shown in Figure 3A, the dPCR chip consists of a three-layer structure, with a glass layer as the substrate, a microchannel and microchamber structure layer in the middle, and a tape layer on top. There were 10,240 microchambers in the chip. The shape of the chambers was a square and the width of the chambers was 100 μm. The depth-to-width ratio of the microchambers was 1:1. The chip has two inlets and one outlet with a diameter of 0.8 mm, 0.8 mm, and 1.6 mm, respectively. Additionally, the evaporation must be considered in the dPCR. The rapid water vapor transport during the thermocycling process makes the reaction solution in the microchambers very easy to evaporate, resulting in the failure of the amplification [37]. In addition, the air permeability of the PDMS aggravates the process of the evaporation. To solve this problem, a water channel embedded around the microchambers were introduced in the chip [16,38].

The chip has a self-priming property and its principle of loading is mainly through negative pressure. The adhesive tape sealed on the top of the chip provides a relatively airtight environment for the chip. After vacuuming, a pressure difference was formed inside and outside of the chip due to the air permeability of the PDMS. The loading process is shown in Figure 3B. When the pipette tip was inserted into the chip, the reaction liquid quickly entered the microchannels and microchambers and then the microchambers and microchannels were separated by mineral oil, making each microchamber an independent reaction chamber. The whole loading process only takes 180 s. Figure 3C shows a picture of the results of the digital PCR reaction solution being injected into the chip. The microchambers were all filled with the reaction solution and the oil phase completely separated the microchambers and the channels.

### 3.4. Quantitative Performance of the Chip

**Figure 4:**
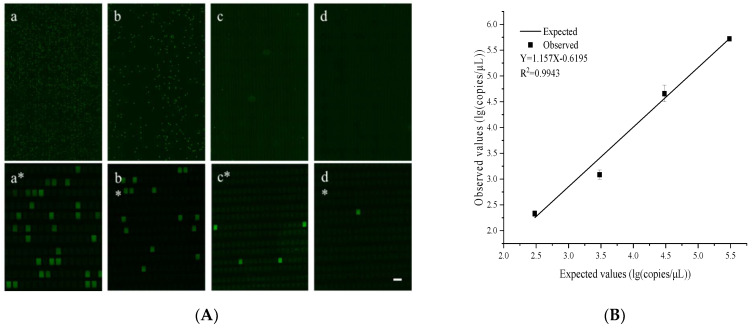
The performance of the developed chip for *S. typhimurium* detection. (**A**) Pictures **a**–**d** are fluorescence images of the digital PCR for a serial dilution of the *invA* genes. Pictures a*–d* are the partial enlarged pictures of the **a**–**d** pictures. (**B**) The linear correlation between the expected values and the observed values. The error bars represent the standard deviation of three replications. The scale bar is 200 μm.

The quantitative performance of the dPCR chip was evaluated using plasmids with a *S. tphimurium invA* gene fragment at different concentrations. The chip can theoretically analyze a single sample in a dynamic range of 4–5 orders of magnitude. Serial solutions of the *S. tphimurium invA* plasmid at 15,000, 1500, 150, and 15 copies/μL were examined under identical conditions. After the PCR thermocycling, the fluorescence images were captured using a fluorescence microscope (emission: 518 nm; excitation: 494 nm; Fam) and then the concentration of the *invA* templates was calculated according to the Poisson distribution [38]. The detailed calculation of the sample concentration is shown in the supplementary information section (S1). The fluorescence images corresponding to each *invA* concentration are shown in Figure 4A They clearly showed that the number of fluorescent microchambers increased proportionally with the *invA* concentration. Additionally, the observed and expected values showed a good linear correlation (R^2^ = 0.9943) with a slope of 1.157 (Figure 4B). These results clearly showed that the chip we designed had a good quantitative performance and was suitable for the dPCR reaction.

### 3.5. Detection of S. typhimurium by the Proposed Platform

**Figure 5:**
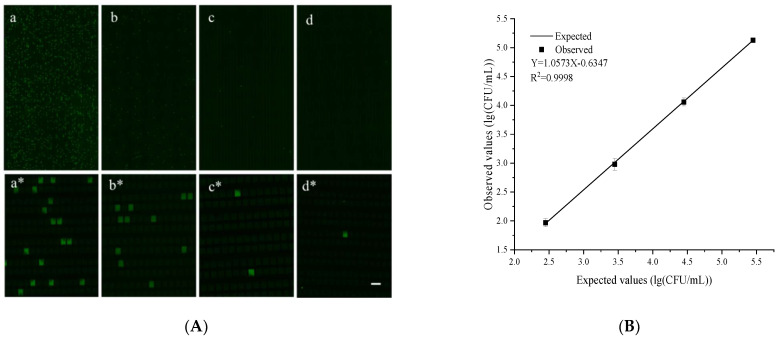
*S. typhimurium* detection by the developed platform. (**A**) Picture **a**–**d** are the fluorescence images of the digital PCR for a serial dilution of *S. typhimurium* in pork samples. Picture a*–d* are the partial enlarged pictures of the **a**–**d** pictures. (**B**) The linear correlation between the expected values and the observed values. The error bars represent the standard deviation of three replications. The scale bar is 200 μm.

To further evaluate the applicability of the developed platform for the detection of *S. typhimurium* in real samples, the prepared *S. typhimurium* solutions spiked in pork matrices with different concentrations from 10^2^ CFU/mL to 10^5^ CFU/mL were used. The subsequent dPCR reactions were carried out after the process of the Apt-MBs capture. Figure 5A shows the fluorescence images of the dPCR after the Apt-MBs capture. They clearly showed that the number of fluorescent microchambers increased proportionally with the initial concentration of *S. typhimurium* in the pork matrices. Additionally, the observed and expected values showed a good linear correlation (R^2^ = 0.9998) with a slope of 1.0573 (Figure 5B). These results showed that the developed platform worked well for the detection of the *S. typhimurium* cells in complex food substrates. However, due to the limitations of the capture efficiencies and subsequent DNA losses, there was a certain error between the digital PCR results and the initial concentrations in the whole process. The observed values were lower than the expected values. However, the platform we developed realized the direct quantification of bacteria without the standard curve, which is of great significance for food and clinical rapid diagnosis. Dehghani et al. [39] developed an aptamer-modified magnetic beads and a LAMP-based method to capture and detect *S. typhimurium* cells in food samples. However, their method had lower capture efficiencies (lower than 80%) and their method can only detect bacteria qualitatively. Our method has higher capture efficiencies in complex food matrices and could realize the quantitative detection of *Salmonella*. Furthermore, our method can detect as low as 90 CFU/per reaction.

### 3.6. Detection of S. typhimurium in Real Food Samples

**Figure 6:**
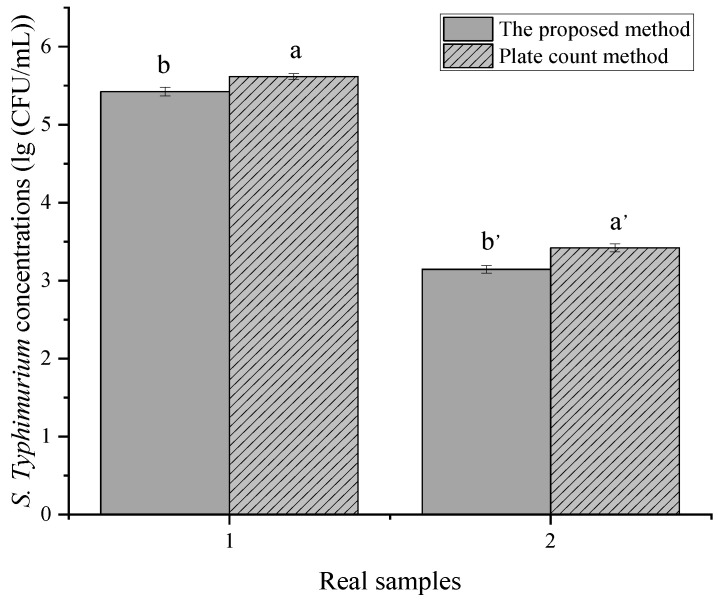
Results of the *S. typhimurium* determination in real samples by the developed method and the plate count method. The error bars represent the standard deviation of three replications.

Unknown concentrations of *S. typhimurium* cells were added to the meat samples to evaluate the quantification of the developed platform. The results are shown in Figure 6. Obviously, this method performed well in meat samples. However, the amounts of bacteria obtained by the method were lower than that by the plate count method due to the limitations of the capture efficiencies, the loss of the DNA extraction, and the matrices of food samples. Nevertheless, our method can still provide a relatively accurate absolute quantitative method and can detect as low as 90 CFU per reaction. Furthermore, when considering the loss of the capture efficiency of the proposed method, there was no significant difference between the two methods. The whole process only takes about 2 h, which greatly shortens the detection time. Compared with other methods, our method can be quantified directly without the construction of a standard curve [8,39]. Although the high background of different types of inhibitors, normal microbiota, and microflora may interfere with bacterial capturing [40], the developed method was sensitive enough to detect and quantify *S. typhimurium* in meat samples because of the highly sensitive dPCR technology.

## 4. Conclusions

In brief, the platform consisting of aptamer-based magnetic beads and a digital PCR chip was demonstrated in the present study to detect *S. typhimurium* in food matrices at low concentrations. The aptamer-based magnetic separation enables the specific recognition of target bacteria. The Apt-MBs could enrich the *S. typhimurium* from pork samples with 94.5% capture efficiencies. The absolute quantification of the captured bacterial cells was achieved by digital PCR on a self-priming microfluidic chip. As little as 90 CFU/per reaction of *S. typhimurium* could be detected and quantified by this method within 2 h from the complex food matrices. This platform provided a rapid, sensitive, and selective assay for the quantification of *S. typhimurium* in complex food samples. Furthermore, the developed platform realized the combination of a pre-chip enrichment of the bacterial cells with an on-chip digital quantification for on-site monitoring of *S. typhimurium* in complex food samples, which is meaningful for point-of-care testing. Additionally, the developed platform can be extended easily to detect other bacteria by employing the corresponding aptamers and probes.

## Supplementary Materials

The following supporting information can be downloaded at: https://www.mdpi.com/article/10.3390/bios12070458/s1, Figure S1: Schematic illustrations of the chip fabrication process; Supplemental method section S1; Supplemental results section S2.
