# Supplementary material for: A Specific and Sensitive Aptamer-Based Digital PCR Chip for Salmonella typhimurium Detection
Source: Biosensors (Basel). 2022 Jun 26;12(7):458. doi: 10.3390/bios12070458 (PMC9313467; doi:10.3390/bios12070458)

biosensors

MDPI

Article

A specific and sensitive aptamer based digital PCR chip for *Salmonella* detection

Yuanjie Suo [1], Weihong Yin [2], Qiangyuan Zhu [1,*], Wenshuai Wu [2], Wenjian Cao [1] and Ying Mu [1,*]

[1] Research Centre for Analytical Instrumentation, Institute of Cyber-Systems and Control, State Key Laboratory of Industrial Control Technology, Zhejiang University, Hangzhou 310027, China
[2] College of Life Sciences, Zhejiang University, Hangzhou 310058, China
* Correspondence: qyzhu2008@zju.edu.cn (Q.Z.); muying@zju.edu.cn (Y.M.)

Supplemental method section S1

For our digital PCR microfluidic chip, the ideal result is that one microchamber is occupied with one or none molecule. However, when the sample was loaded into the chip, the DNA molecules were randomly and independently distributed to microchambers. The one-to-one or one-to-none pattern cannot be guaranteed. The process of sample partition was fitting Poisson distribution. The concentration of samples can be calculated according to the Poisson distribution formula:

$$P(n,\lambda) = (\lambda^n \cdot e^{-\lambda})/n! \qquad (1)$$

Where n is the number of DNA molecules in microchamber (0, 1, 2, 3…) and λ is the average number of DNA molecules in microchambers, P means the probability that there are n molecules in microchambers. After the process of thermocycling, the microchambers contained at least one DNA molecule would show positive signals (green fluorescence), thus

$$\mathrm{P(n > 0) = 1 - P(n = 0)} = 1 - e^{-\lambda} \qquad (2)$$

The chance of having templates in microchambers also equals the proportion of positive microchambers, then

$$P(n > 0) = 1 - e^{-\lambda} = positive/total \qquad (3)$$

positive represents the count of positive microchambers and *total* is the amount of microchambers on chip. When there are *X* DNA molecule in the developed chip,

$$\lambda = X/total$$

The formula (3) can be rewritten as:

$$1 - e^{-X/\mathrm{total}} = positive/total \qquad (4)$$

Or

$$X = ln(1 - positive/total) \cdot (-total) \qquad (5)$$

Furthermore, the volume of the single microchamber and total microchambers can be calculated from the known size of microchamber.

Thus, the concentration of the loading sample can be calculated by counting the positive chambers in chip and Poisson distribution formula.

Supplemental results section S2

A pre-experiment was conducted to determine the starting point of optimization of capture efficiency. When the amount of Apt-Mbs was 40 μL, there was only about 64.5% of the capture efficiency. When the amount of Apt-Mbs was 60 μL, there was about 79.2% of the capture efficiency. All the capture efficiencies were below 85%. However, the capture efficiency could reach to a high level (~ 90%) when the amount of Apt-Mbs was 80 μL. The above experimental results showed that as the concentration increased, the capture efficiencies increased. In order to improve the capture efficiency, we finally settled on 80 μL as the starting point for the optimization experiment.

Figure S1: Schematic illustrations of the chip fabrication process.

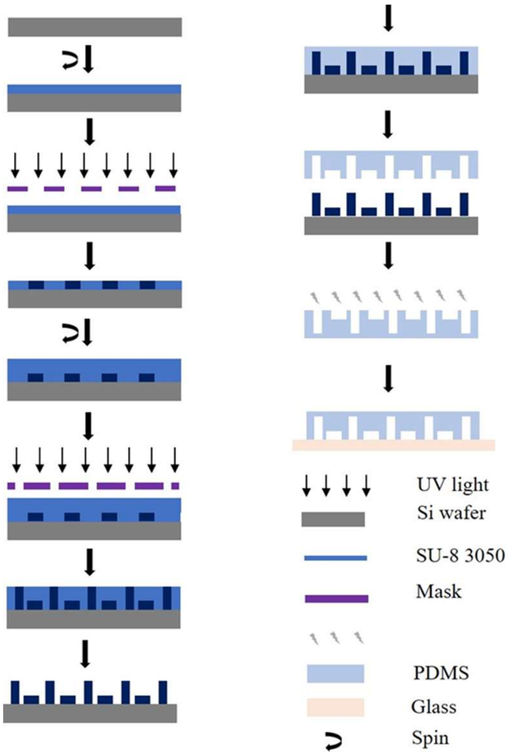

Supplement: Supplementary file 1 [file biosensors-12-00458-s001.zip › biosensors-1761451-supplementary.pdf]
